# The Association between Pre-existing Diabetes Mellitus and Pressure Ulcers in Patients Following Surgery: A Meta-analysis

**DOI:** 10.1038/srep13007

**Published:** 2015-08-11

**Authors:** Zhou-Qing Kang, Xiao-Jie Zhai

**Affiliations:** 1 Department of Nursing, Jinqiu Hospital of Liaoning Province, 317 Xiaonan Street, Shenhe District, 110016 Shenyang, China

## Abstract

Uncertainty exists about the role of diabetes in the development of surgery-related pressure ulcers. Therefore, we conducted a meta-analysis to explore the association between pre-existing diabetes mellitus and pressure ulcers among patients after surgery. Summary odds ratios (ORs) and 95% confidence intervals (CIs) were calculated using random effects models. Thirteen eligible studies of 2367 patients in total and 12053 controls were included in the final analysis. Compared with patients without diabetes, the pooled odds ratio (OR) of the incidence of pressure ulcers in diabetic patients was 1.74 [95% confidence interval (CI) = 1.40–2.15, I^2 ^= 51.1%]. Estimates by type of surgery suggested similar results in cardiac surgery [OR = 2.00, 95% CI = 1.42–2.82, I^2 ^= 0%], in general surgery [OR = 1.75, 95% CI = 1.42–2.15, I^2 ^= 0%], and in major lower limb amputations [OR = 1.65, 95% CI = 1.01–2.68, I^2 ^= 0%] for diabetic patients versus non-diabetic controls. We did not find an increased incidence of pressure ulcers in diabetic patients undergoing hip surgery compared with non-diabetic controls [OR = 1.46, 95% CI = 0.62–3.47, I^2 ^= 93.1%]. The excess risk of pressure ulcers associated with pre-existing diabetes was significantly higher in patients undergoing surgery, specifically in patients receiving cardiac surgery. Further studies should be conducted to examine these associations in other types of surgery.

Pressure ulcers have become a common problem faced by global health care institutions and seriously threaten the life and health of the patient, leading to large economic pressures and societal health burdens. Therefore, accurately identifying patients who are at risk of developing pressure ulcers is the key to prevention. Patients undergoing surgical procedures are a high-risk group for pressure ulcers; reported incidence rates of perioperative pressure ulcers have ranged from 3.4% to 66%^2^,^3^,^4^,^5^,^6^,^7^,^8^,^9^,^10^,^11^,^12^,^13^,^14^.

Surgical risk factors leading the development of pressure ulcers include the number of surgeries, total time in the operating room, surgical site, and the use of extracorporeal circulation, among other factors^2^,^3^,^9^,^10^,^14^. One possible reason may be that the patient is immobile for long periods during surgical procedures and is unable to relieve the pressure on bony prominences. Consequently, tissue ischemia results because of capillary blood flow occlusion from prolonged unrelieved pressure. In addition to the aforementioned surgical risk factors, diabetes mellitus is also a characteristic thought to be commonly associated with perioperative pressure ulcers^2^,^3^,^4^,^5^,^6^,^7^,^8^,^9^,^10^,^11^,^12^,^13^.

To date, several studies have focused on the association between diabetes mellitus and surgery-related pressure ulcers; however, these published reports have varied by incidence, type of surgery, and risk factors, among other reasons. A previous meta-analysis conducted by Liu *et al*.^15^ examined the association and calculated the total efficacy rate but did not include all eligible publications. In addition, the conclusions were not stratified by different types of surgery. Furthermore, many new relevant cohort and case-control studies have now been published, especially three recently published studies^1^,^2^,^4^ that each involved large samples of more than two thousand participants. Thus, we undertook an updated and extended analysis that incorporated additional previous and more recent data to further assess diabetes as a risk factor for pressure ulcers in patients undergoing different types of surgery.

## Methods

### Search strategy

A comprehensive literature search was performed using the PubMed (1946-October 2014) and EMBASE (1947-October 2014) electronic databases by two independent investigators (ZQK and XJZ). The following search strategy, adapted for PubMed and EMBASE, was used for the searches without restrictions: (“operative” OR “operation” OR “surgery” OR “surgical” OR “surg*”) AND (“diabetes” OR “diabetic” OR “Diabetes Mellitus” OR “diabet*”) AND (“pressure ulcer” OR “bedsore*” OR “pressure sore*” OR “bed sore*” OR “decubitus ulcer*” OR “bed-sore*”). Furthermore, we conducted a manual search by checking the cited reference lists of the original reports to locate additional relevant studies. Unpublished reports were not considered.

### Study selection

The titles or abstracts of all of the identified studies were screened by two independent reviewers (ZQK and XJZ). The full text was retrieved for further assessment when the reviewers could not evaluate a study’s topic from its title or abstract. Discrepancies were resolved by discussion. The inclusion criteria for screening the studies were as follows: (1) original human studies published in English; (2) original epidemiologic studies (i.e., RCT, cohort or case-control); (3) pre-existing diabetes mellitus was the exposure; (4) the outcome was the development of pressure ulcers; (5) the study investigated the association between pressure ulcer and pre-existing diabetes mellitus among perioperative patients; and (6) risk estimates (odds ratio or relative risk) were published along with their 95% confidence intervals (CI) or enough data were provided to calculate these estimates. Studies were excluded if they (1) were letters, comments, correspondences, review articles or case reports; (2) were based on small sample size (<30 patients); (3) provided insufficient data; or (4) did not examine relevant outcomes. If multiple studies were found to share an identical population, we only included the most recent publication.

### Data extraction and quality assessment

Two reviewers (ZQK and XJZ) independently extracted the data from the eligible studies using piloted and standardized data extraction forms. The form included the first author’s name, publication year, type of publication, study geographic location, study design, inclusion period, operation methods, sample size, mean/median age, diabetes type, treatment regimen, use of multivariate logistic model analysis, follow-up period, adjustment factors, and ORs with corresponding 95% CIs. Discrepancies were resolved by discussion.

The eligible studies were assessed by two independent reviewers (ZQK and XJZ) using the Newcastle-Ottawa Scale (NOS)^16^. The quality of each study was evaluated using 3 major categories: selection, comparability and exposure/outcomes. A full NOS score was 9 stars; an awarded score of 5 stars or more was defined as a high-quality research in our study according to standards of previous meta-analysis^17^.

### Statistical analysis

The meta-analysis was performed using STATA statistical software (version 12.0, Stata Corporation, College Station, TX, USA). We retrieved or calculated the OR estimates with a 95% CI from the baseline form. Summary ORs and 95% CIs were performed using a random effects model due to the potential heterogeneity among the studies in the meta-analysis (e.g., methods of surgery, study designs, follow-up time, etc.). Inter-study heterogeneity was explored and quantified using the I^2^ test; an I^2 ^> 50% indicated significant heterogeneity^18^. Reasons for heterogeneity were detected through sensitivity analyses. Publication bias was assessed visually by inspecting funnel plots and by using Egger’s or Begg’s regression test whereby a P-value < 0.10 was considered to be significant^19^.

We performed subgroup analyses to reveal potential associations among the different types of operations (e.g., hip surgery, major lower limb amputations, cardiac surgery and general surgery).

## Results

### Search results and study characteristics

**Figure 1:**
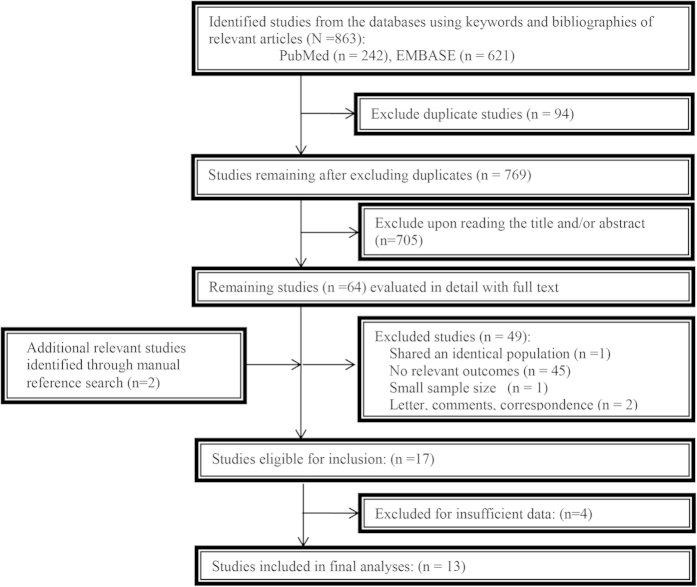
The flow diagram of the screened, excluded, and analyzed publications.

A total of 863 studies were identified from the PubMed and EMBASE electronic databases; of these, 64 studies were considered to have potential value for further review. We retrieved the full texts for detailed evaluation and identified two relevant individual studies through a manual reference search. By excluding unrelated studies based on the inclusion criteria, 17 total studies were identified. Lastly, we excluded 4 studies with insufficient data when we could not acquire the necessary information by contacting the authors directly. Finally, thirteen eligible studies were included in our meta-analysis. The screening process was summarized in a flow diagram (Fig. 1).

Table 1 outlines the characteristics of the 13 included studies, totaling 2367 patients and 12053 controls including 1422 incident perioperative pressure ulcer events during the follow-up periods. All of the selected studies were observational, including eight prospective cohort studies and three retrospective case-control studies. Seven studies were from the US, two from the UK, and one each was from Sweden, Italy, Spain and Belgium. Logistic regression analysis was the most common approach, used in nine of the eligible studies. The remaining four studies were performed using univariate analysis. The data collection period was from 1995 to 2009 (five studies did not report the data collection period). All of the eligible studies were considered to be of high quality, ranging from five to eight points according to the Newcastle-Ottawa Scale (Table 1).

### Results of meta-analysis

**Figure 2:**
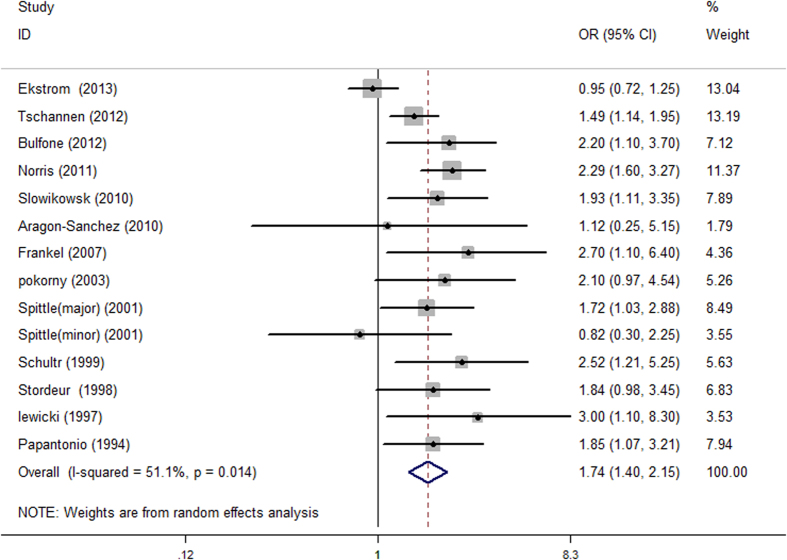
The forest plot comparing the association between diabetes mellitus and the risk of perioperative pressure ulcers.

We conducted a primary meta-analysis using all thirteen studies included in the final analysis. The pooled summary OR of pressure ulcer incidence in diabetic patients was 1.74 [95% CI = 1.40–2.15, I^2 ^= 51.1%] compared with individuals without diabetes (Fig. 2).

**Figure 3:**
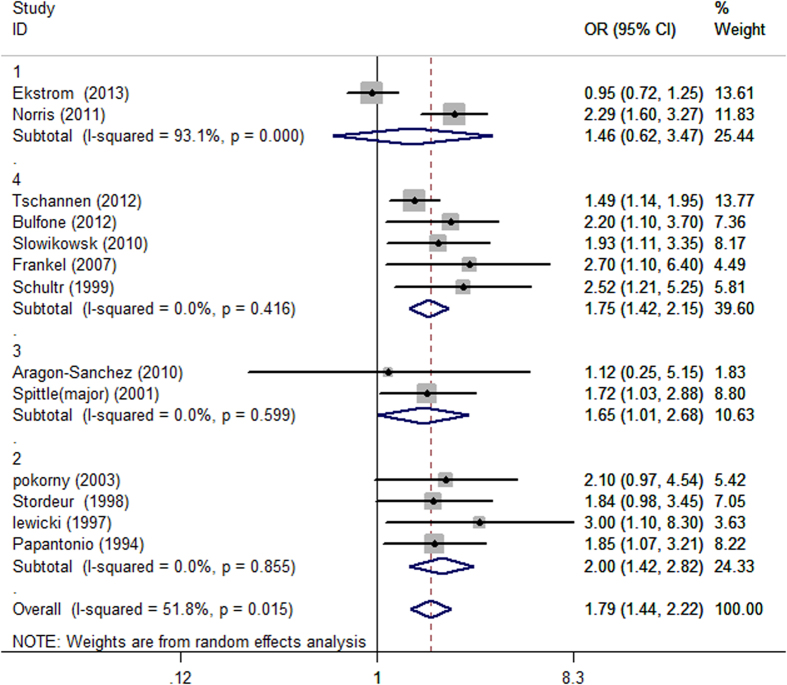
Forest plot of the subgroup analyses stratified by surgery type.

We conducted subgroup analyses according to the surgical method in order to further explore the associations between diabetes and the risk of perioperative pressure ulcers (Fig. 3). The subgroup analysis of four studies examining cardiac surgery suggested a significant association [OR = 2.00, 95% CI = 1.42–2.82, I^2 ^= 0%]. Similar results were also found in the general surgery subgroup [OR = 1.75, 95% CI = 1.42–2.15, I^2 ^= 0%] and in the major lower limb amputation subgroup [OR = 1.65, 95% CI = 1.01–2.68, I^2 ^= 0%]. We did not observe an increased incidence of pressure ulcers between diabetic patients undergoing hip surgery compared with non-diabetic controls [OR = 1.46, 95% CI = 0.62–3.47, I^2 ^= 93.1%].

### Test of heterogeneity and sensitivity analyses

**Figure 4:**
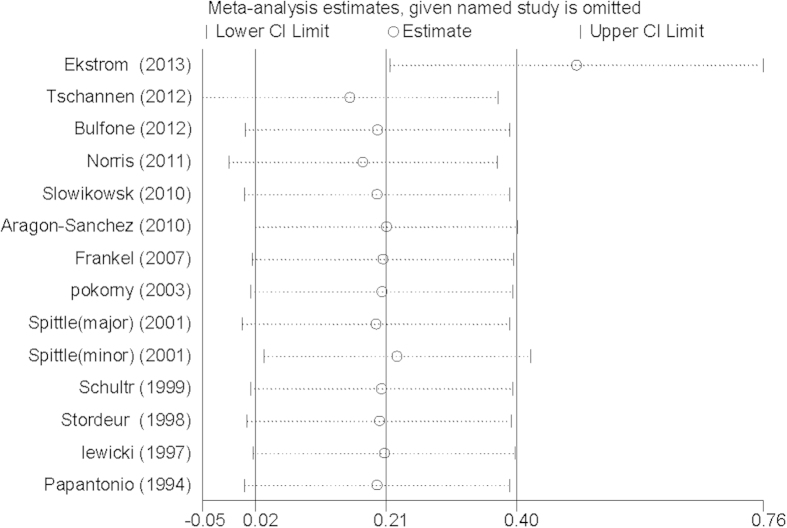
Sensitivity analysis using a random effects model of the logit dropout rate.

There was significant heterogeneity among the eligible studies [P = 0.014, I^2 ^= 51.1%]. In order to detect the possible reasons for heterogeneity, we used the leave-one-out sensitivity analysis technique. When removing the study by Ekstrom *et al*.^1^, the estimate using all of the other studies was clearly altered [OR = 1.63, 95% CI = 1.25–2.14] (Fig. 4).

### Publication bias

**Figure 5:**
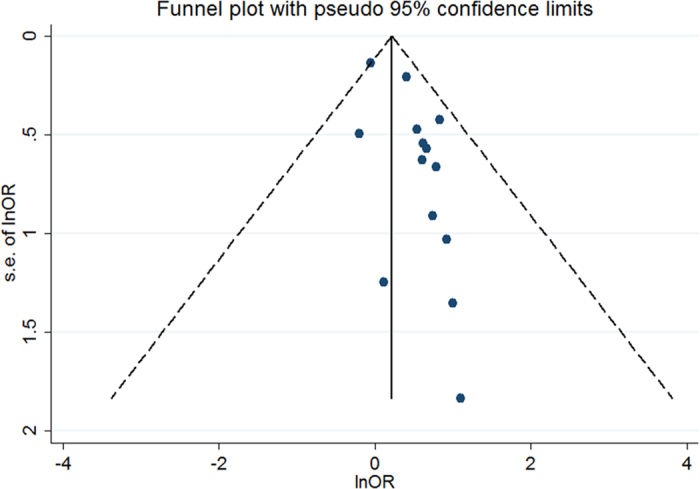
Begg’s funnel plot examining the publication bias of diabetes mellitus and the risk of perioperative pressure ulcers.

The funnel plot showed slight asymmetry (Fig. 5), and possible publication bias existed among the 13 included studies (Begg’s test, P for bias = 0.228; Egger’s test, P for bias = 0.009).

## Discussion

Our updated meta-analysis suggested that diabetes mellitus may lead to a higher risk of perioperative pressure ulcers. In subgroup analyses, similar results were found in cardiac surgery, general surgery and major lower limb amputations.

Among the diabetic patients undergoing cardiac surgery, we found that the risk of pressure ulcers was twice that for the non-diabetic controls. Compared with other types of surgery, restricted movement from cardiac assistive devices (e.g., intra-aortic balloon pumps, left ventricular assist devices, etc.) and heart failure were considered to be contributing factors for the pressure ulcers in patients undergoing cardiac surgery^8^.

For lower limb amputations, diabetes mellitus has been widely considered to be a risk factor^20^ compared with amputees without diabetes; the presence of diabetes often indicated a worse prognosis and a higher incidence of pressure sores^6^,^21^. Peripheral neuropathy in diabetic patients might assist the development of pressure ulcers due to injuries to protective pain sensations and interferences with micro-vascular circulation^9^.

No significant association was observed in the hip surgery subgroup. The subgroup analysis of the 5966 hip surgery cases^4^ indicated that patients with diabetes had a higher incidence and risk of pressure ulcers than the non-diabetic group. However, the other included study of 2133 patients^1^ did not indicate a clear difference in the incidence of pressure ulcers between diabetics and non-diabetics. The most likely explanations could be that 76% of the participants in Ekstrom’s study^1^ had ASA class (American Society of Anesthesiologists’ classification) 3–5, indicating entirely reduced physical activity and health. This finding may have led to the inconsistent results from the two studies.

Compared with the prior meta-analysis of the six studies conducted by Peng Liu *et al*.^15^, we included thirteen studies and performed more subgroup analyses to explore the potential confounders that influenced the findings. We conducted more powerful and detailed analyses to obtain our results. First, we included more studies in the analysis, especially three recently published studies that each had large sample sizes exceeding two thousand participants. Second, we conducted more comprehensive subgroup analyses. Despite the similar results found among cardiac surgery and general surgery patients, we also found a consistency effect among patients with major lower limb amputations.

Several limitations in our meta-analysis should be noted. First, we only searched for studies published in English that were included in PubMed and EMBASE; thus, some relevant studies published in other languages and in additional databases may not be identified in our research. Moreover, caution should be warranted when interpreting the overall study estimates because there was significant heterogeneity. Furthermore, we did not register our meta-analysis at inception. We suggest that future systematic reviews should be prospectively registered to improve transparency in the review process and prevent selective publication bias.

In summary, the excess risk of pressure ulcers associated with diabetes is significantly higher in participants undergoing surgery, specifically in patients receiving cardiac surgery. Further studies should be conducted to assess the association in other types of surgery.

## Additional Information

**How to cite this article**: Kang, Z.-Q. and Zhai, X.-J. The Association between Pre-existing Diabetes Mellitus and Pressure Ulcers in Patients Following Surgery: A Meta-analysis. *Sci. Rep*. **5**, 13007; doi: 10.1038/srep13007 (2015).

## Figures and Tables

**Table 1:** Characteristics of the identified studies.

First author, year(reference)	Country	Study design	Study period	Surgery type	Sample size	Mean/median age(years)	Adjusted factors	NOS scores: Selection/comparability/outcome(exposure)
Ekstrom, 2013^1^	Sweden	Prospective cohort study	NA	hip fracture	2133 (DM:234)	DM: 82 (SD 8.5); non-DM:81 (SD 10.8)	American society of anesthesiologists’ classification, walking ability(one walking aid/walking frame), comorbidity (cardiovascular/cerebrovascular lesion/kidney disease), hip pain before fracture	4/0/3
Tschannen, 2012^2^	USA	Prospective cohort study	2007.11 to 2009.08	general surgery	3225(DM:763)	58.9 (SD 16.0)	Age, body mass index, total time in operating room, maximum time in operating room, braden score on admission, use of vasopressors, number of surgeries, risk of mortality	3/1/2
Bulfone, 2012^3^	Italy	Prospective cohort study	2009.09 to 2009.10	general surgery	102(DM:14)	62.3 (SD 14.3)	NA	4/0/3
Norris, 2011^4^	UK	Prospective cohort study	1989.01 to 2008.10	hip fracture	5966 (DM:477) (DM-1:99; DM-2:378)	DM-1: 75; DM-2: 79.8; non-DM:80	Age, using walking aids, mean mobility score,	4/1/3
Slowikowski, 2010^5^	USA	Prospective cohort study	2005.03 to 2008.05	general surgery	369(DM:87)	58.3 (SD 19.3)	Age, Braden Scale score	3/1/2
Aragon-Sanchez, 2010^6^	Spain	Retrospective case-control study	1998.01 to 2008.12	Major Lower limb Amputations	283(DM:221)	DM: 73; non-DM:78	Age, heart disease, dislipidemia, high blood pressure, previous amputation, time from the previous major Amputation,	3/1/2
Frankel, 2007^7^	USA	Retrospective case-control study	NA	general surgery	820(DM:147)	57.7	High blood urea nitrogen, high creatinine, vascular disease, spinal cord injury	3/0/2
Pokorny, 2003^8^	USA	Prospective cohort study	1997 to 1998	cardiac surgery	351(DM:117)	63.6	Age, gender, time from admission to surgery, time from admission to hospital discharge	4/1/3
Spittle, 2001^9^	UK	Retrospective case-control study	1995.01 to 1998.12	Lower limb amputations	122(DM:67)	DM:70.6; non-DM:73.2	Age	3/1/2
Schultz, 1999^10^	USA	Prospective cohort study	NA	general surgery	413(DM:95)	65.7	Age, body mass index, admit Braden Scale score, Surgical procedure,	4/1/3
Stordeur, 1998^11^	Belgium	Prospective cohort study	1995.03 to 1995.05	cardiac surgery	163(DM:30)	64.5 (SD 11.3)	Hemoglobin, length of stay, Norton score and Braden score at admission, postoperative Norton score and Braden score	3/0/3
Lewicki, 1997^12^	USA	Prospective cohort study	NA	cardiac surgery	337(DM:87)	62 (SD 11.59)	Lower hemoglobin, hematocrit, serum albumin levels, greater comorbidity, time required to return to preoperative body temperature, being turned only once a day, presence of an intra aortic balloon pumps	4/0/3
Papantonio, 1994^13^	USA	Prospective cohort study	NA	cardiac surgery	136(DM:28)	61.9	Age, albumin, hematocrit,	3/1/2

Abbreviations: NA, not available; DM, Diabetes Mellitus; DM-1, Type 1 diabetes mellitus; DM-2, Type 2 diabetes mellitus; SD, standard deviation; NOS scores, the study’s scores of quality assessed by the Newcastle-Ottawa Scale.
